# Utilization of Palm Oil Clinker as Cement Replacement Material

**DOI:** 10.3390/ma8125494

**Published:** 2015-12-16

**Authors:** Jegathish Kanadasan, Hashim Abdul Razak

**Affiliations:** StrucHMRS Group, Department of Civil Engineering, Faculty of Engineering, University of Malaya, Kuala Lumpur 50603, Malaysia; jegathish@siswa.um.edu.my

**Keywords:** palm oil clinker, palm oil clinker powder, self-compacting mortar, sustainability

## Abstract

The utilization of waste materials from the palm oil industry provides immense benefit to various sectors of the construction industry. Palm oil clinker is a by-product from the processing stages of palm oil goods. Channelling this waste material into the building industry helps to promote sustainability besides overcoming waste disposal problems. Environmental pollution due to inappropriate waste management system can also be drastically reduced. In this study, cement was substituted with palm oil clinker powder as a binder material in self-compacting mortar. The fresh, hardened and microstructure properties were evaluated throughout this study. In addition, sustainability component analysis was also carried out to assess the environmental impact of introducing palm oil clinker powder as a replacement material for cement. It can be inferred that approximately 3.3% of cement production can be saved by substituting palm oil clinker powder with cement. Reducing the utilization of cement through a high substitution level of this waste material will also help to reduce carbon emissions by 52%. A cleaner environment free from pollutants can be created to ensure healthier living. Certain industries may benefit through the inclusion of this waste material as the cost and energy consumption of the product can be minimized.

## 1. Introduction

**Figure 1:**
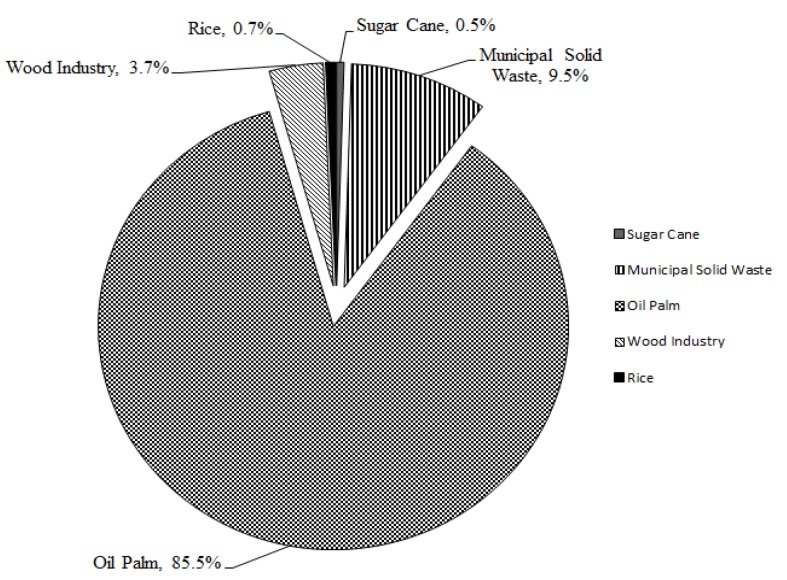
Biomass produced by different industries in Malaysia (Adapted from Shuit *et al.* [8]).

The utilization of waste by-products in concrete has garnered positive outcomes over the past few decades in terms of the cost savings and conservation of natural resources. Some of the resources currently being employed for concrete production are prone to having negative effects on the environment besides being non-renewable. This has resulted in an increase in research to develop alternative feed to reduce and maintain a non-excessive usage of natural sources. The agricultural industry in Malaysia has developed progressively over the past few decades, substantially supporting the economy of the country. The industry has diversified its product output from the basic fresh products up to completely processed goods. Concurrently, a huge amount of waste by-products are also produced during the manufacturing stages which need serious consideration. Eighty million tonnes of dry solid biomass waste was yielded in 2010 by the oil palm industry in Malaysia and is expected to rise up to 85–110 million tonnes by 2020 [1]. In depth research and studies carried out on these waste materials could increase the chances of utilizing or recycling this material again in another industry and thereby reduce the continuous exploitation and conserve the available natural resources for use in future. Around 57 million tonnes of oil was made by Malaysia and Indonesia together by 2012, which makes up 85% of the overall global palm production [2,3]. The palm oil industry in Malaysia plays an integral part of the country’s economic growth. Statistics show that there are 440 fresh fruit bunch (FFB) mills in Malaysia spread throughout Peninsular Malaysia, Sabah and Sarawak [4]. Figure 1 depicts different types of biomass produced by various industries in Malaysia. Table 1 tabulates the types of biomass obtained from palm oil mill processing stages and the quantity produced. Malaysian Palm Oil Board (MPOB) [5] reported that 19.22 million tonnes of crude palm oil (CPO) was produced in 2013 in Malaysia which was 2.3% higher than the previous year. Concurrently, the amount of waste biomass generated from the palm oil mill industry is also expected to be increasing proportionally whereby there will be a need for proper waste management system to avoid serious environmental pollution. Sumathi *et al.* [6] reported that the small oil content within mesocarp fibre (MF) and shell can be utilized as a fuel to produce steam for the mill’s operation. In addition, incineration of shell and fibre in the boiler generates steam which is utilized in CPO production and for some of the electricity for the mill’s consumption [6]. Vijaya *et al.* [7] reported that, on average, approximately 0.05 tonne of boiler ash is produced for every tonne of CPO when MF and shell are incinerated in the boiler. Thus, taking into account the amount of MF and shell produced annually by oil palm mills, production of boiler ash could be also rising proportionally which increases the need for proper waste disposal and management systems.

**Table 1:** Types of biomass and quantity produced (Adapted from Sumathi, Chai and Mohamed [6]).

No.	Type of Biomass	Quantity/Annum (Mt)
1	Empty fruit bunch (EFB)	15.8
2	Fronds	12.9
3	Mesocarp fibre (MF)	9.6
4	Trunk	8.2
5	Shell	4.7

Palm oil clinker (POC) is a waste by-product gathered after the complete incineration process of oil palm shell and fibre. Physically they are porous, grey in colour, irregular in shape and much lighter. Most of the palm oil processing plants dispose of the clinker in them by using them as a cover for the potholes on the roads within the vicinity of the plantation areas [7]. Rather than utilizing them for some purpose that could harm the environment, it would be better and more ideal to channel them into the construction industry. As POC aggregate is lighter in nature, it can be utilized for the production of lightweight concrete or mortar. This would be a very efficient way to avoid environmental pollution besides benefiting the construction industry as an aggregate replacement. Environmentally friendly and highly energy efficient materials need to be introduced to reduce the environmental pollution arising from the high carbon footprint of cement in its production stages. Substitution with environmentally safer materials is vital to ensure the concrete produces a lower emission factor. There are few studies carried out on POC in the past. A self-compacting concrete using POC showed that almost 68% of the compressive strength can be achieved when POC is replaced with natural aggregates [9]. Moreover, studies performed by Kanadasan and Abdul Razak [10] shows that use of POC aggregates produced concrete with “good” category ultrasonic pulse velocity (UPV) values. Incorporation of oil palm boiler clinker in oil palm shell concrete between 0% and 50% managed to lower down the density of the concrete by 21%–27% [11]. Complete replacement of POC as aggregates reduced the weight of concrete by 16% compared to control specimen [12]. A feasibility study performed on POC utilization in construction industry using samples from all states in Malaysia showed that POC specimens can produce structural efficiency in the range of 0.035–0.05 MPa/(kg/m^3^) which is similar to control specimen [13]. A past study showed that POC only lowered the concrete strength by 13%–31% compared to control specimens [14]. In addition, POC concrete produced satisfactory electrical resistivity values indicating good durability properties [15]. From structural point of view, singly reinforced POC concrete beams which has a reinforcement ratio lower than 0.5% exhibited satisfactory deflection within the acceptable range [16]. Utilization of POC decreased the weight of concrete slabs by 18.3% compared to the normal concrete slabs [17]. Besides that, Mohammed, Al-Ganad and Abdullahi [17] also found from their study that POC slab showed lower structural properties when compared to normal slab as the modulus of elasticity of POC concrete is lower. In a separate study, reinforced POC concrete beam showed similar shear performance as compared to normally reinforced concrete beam [18]. Introducing waste material instead of using other natural resources would be a better way to enhance the sustainability. Considering this situation, POC powder was integrated into the mix proportion to replace cement at various substitution levels, which will lower the emission factor as well as provide an alternative for a proper disposal system. As the amount of natural resources is constant, introducing these by-products ensure their availability to meet future needs.

Recently, the utilization of powder materials in the construction industry has increased. Taking into account the environmental pollution, cement is blended with other waste materials that could improve or enhance the hardened and durability properties. In addition, the cost of the concrete can also be significantly reduced without sacrificing the mechanical performance. Researchers have found that the incorporation of 45% waste concrete powder (WCP) actually increased the sorptivity coefficient by 70% compared to the control [19]. Researchers found that substitution of cement with 30% of municipal solid waste incineration (MSWI) bottom ash has the ability to produce compressive strength of about 38.9 MPa at 28 days which is above the Class 32.5 as specified by Chinese National Standard GB 175-2007 [20]. The incorporation of bamboo leaf ash as a replacement for cement at 10% and 20% produced mortar specimens with a strength loss of 1% and 2.8% compared to that of the control specimens at 28 and 90 days of curing [21]. The addition of class F fly-ash produced lower chloride intrusion results at 90 and 365 days of approximately smaller than 400 and 700 Coulombs, respectively [22]. Researchers have reported that the inclusion of natural pozzolana and marble powder generally showed satisfactory results in terms of the evolution of compressive strength when replaced with cement [23]. Li *et al.* [24] reported that incorporation of dry composite electroplating sludge (CEPS) in decorative mortar showed comparable compressive strength, flexural strength and tensile bond strength properties with respect to control specimens. Cement mixed with 10% of untreated cement kiln dust (CKD) gave good strength properties but further addition produced lower strength [25]. After 90 days of curing, mortar specimens with 20 μm of ground glass showed 2% higher strength evolution to that of the control mix [26]. Throughout the study, mortar samples with 5% of high calcium wood ash (HCWA) produced higher flexural strength compared to the control concrete [27]. Substitution of 20% waste glass with sand in concrete increased both compressive strength and flexural strength at 28 days by 4.23% and 11.20%, respectively, above control samples [28]. When finely ground basaltic ash (NP), limestone powder (LP) and ordinary Portland cement (OPC) were used in the ternary blend with a ratio of 55OPC:15LS:30NP, the chloride ingression could be reduced significantly and would decrease the CO_2_ emissions by 48% [29]. Blending reburnt rice husk ash (RHA) with cement at 30% substitution produced concrete with good reduction in terms of chloride permeation, chloride diffusion and water permeability by about 75%, 28% and 35%, respectively [30]. The replacement of 10% of waste LCD glass powder produced 94%–99% of the compressive strength and 96%–99% of the flexural strength compared to an ordinary Portland cement (OPC) mix [31]. Researchers reported that the compressive strength, flexural strength and splitting tensile strength of lightweight foamed concrete with 10%–20% of palm oil fuel ash (POFA) as filler were higher compared to 100% sand filler lightweight foamed concrete [32]. In addition, incorporation of ground palm oil fuel ash (GPA) for high strength concrete production reduced the water permeability of the concrete which is about half compared to Type 1 Portland cement high strength concrete [33]. It was reported that the use of sugar cane baggase ash (SCBA) managed to reduce the emission of CO_2_ by 519.3 kilotonnes per year [34].

Although studies were carried out on using POC as aggregates in concrete, there is no research work reported till date on using POC powder as a material to supplement cement. Thus, this work would be focused on using POC powder as a replacement material for cement for mortar production. In this study, POC powder was investigated for use as a binder material to replace cement. It was substituted at different levels varying from minimal replacement to maximum replacement to determine the optimum level of replacement. It was obtained by grinding POC into a fine powder form and substituting the cement at different percentages. The replacement levels were maximized at 50% to establish the performance of the specimens subjected to lower cement content. Their fresh and hardened properties tests were evaluated to investigate the effectiveness of POC powder in mortar specimens to replace cement. Several chemical and microstructure tests were carried out to further investigate the characteristics and effects of using POC powder in the mortar specimens. The sustainability aspects of POC powder incorporation was also evaluated both economically and environmentally to understand the positive impacts on the industry and environment. From this study, the effectiveness and feasibility of using POC powder as a replacement material for cement can be obtained. In addition, this research can elevate the sustainability of the construction industry and contribute significantly in respect to replacement binder materials.

## 2. Experimental Programme

### 2.1. Material

**Figure 2:**
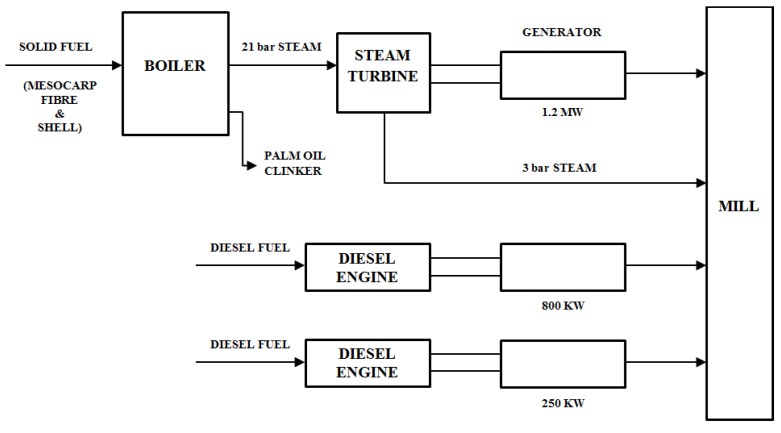
Schematic diagram of a typical power house in palm oil mill (Adapted from Yusoff [35]).

**Figure 3:**
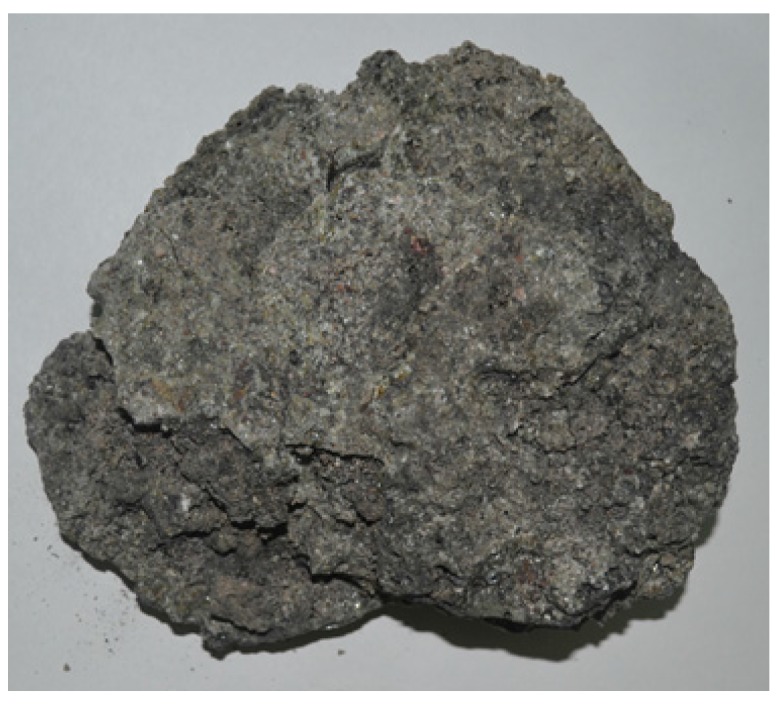
A large piece of POC collected from palm oil mill.

**Figure 4:**
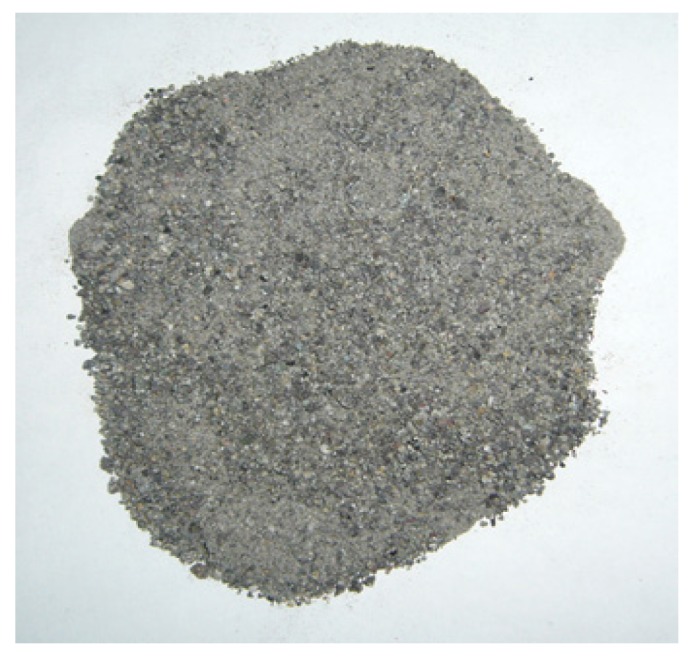
POC fine.

**Figure 5:**
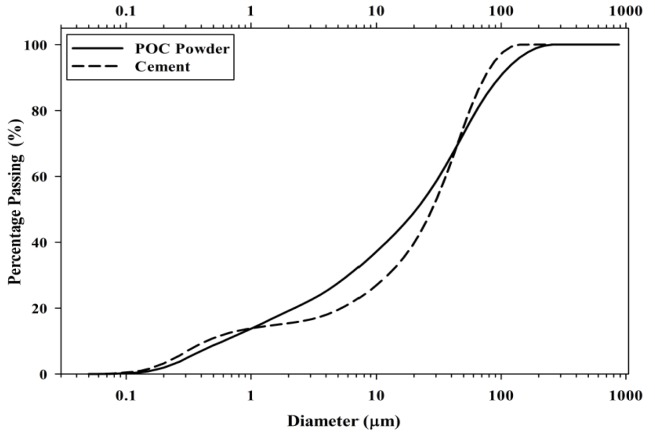
Particle size analysis for POC powder and cement.

**Figure 6:**
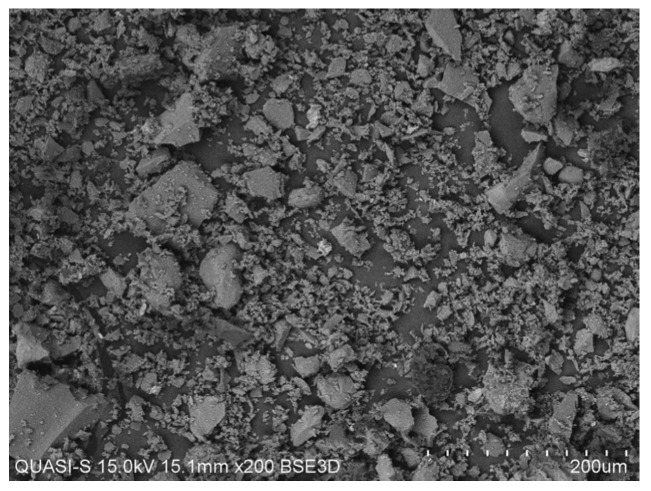
POC powder morphology obtained through SEM test.

**Figure 7:**
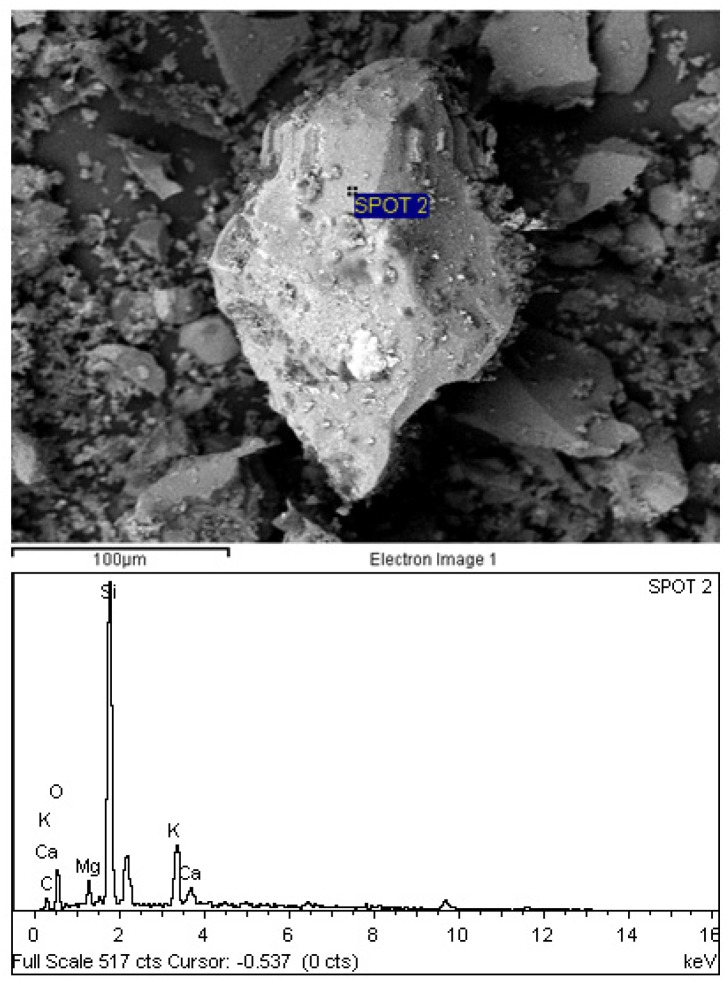
POC powder structure at a smaller magnification with X-ray spectroscopy (EDX).

**Figure 8:**
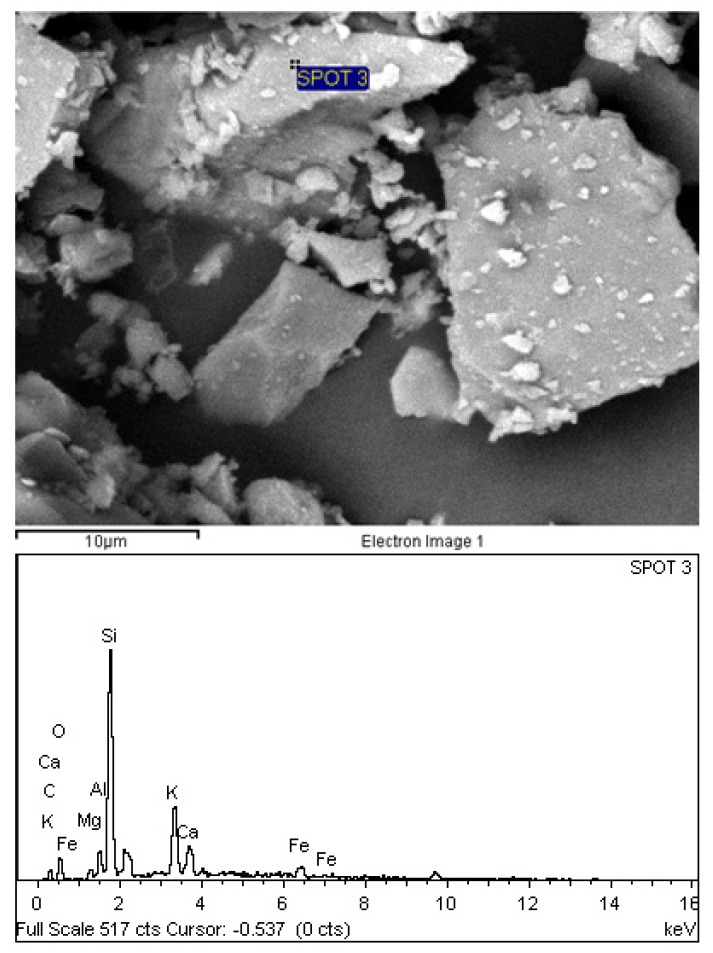
Irregular shape of POC powder specimens with EDX results.

**Figure 9:**
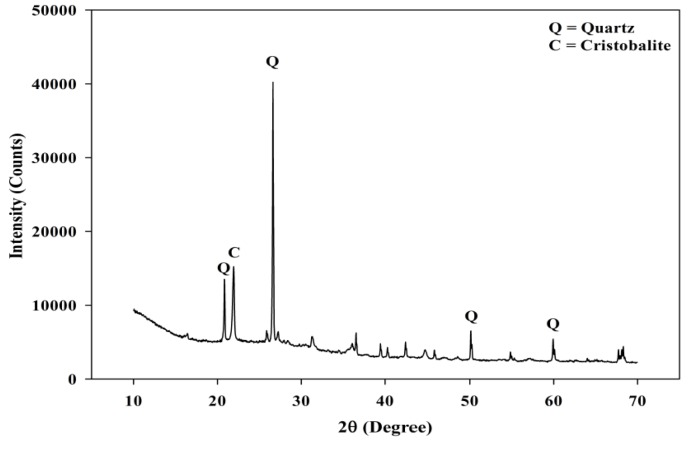
X-ray diffraction (XRD) of POC powder.

**Figure 10:**
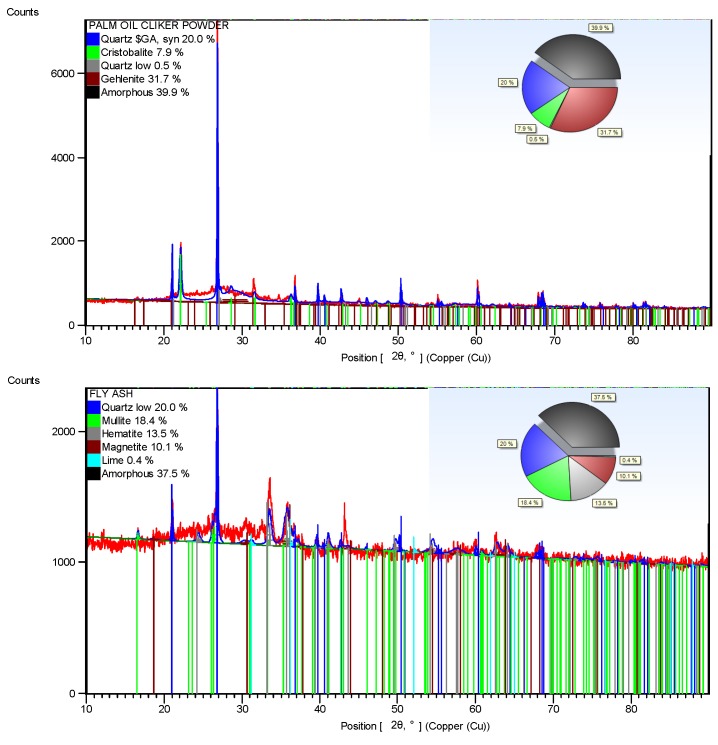
Quantitative XRD results for POC powder and fly ash.

Figure 2 shows the adapted schematic diagram of a typical power house in a palm oil mill. As aforementioned, POC is obtained from the oil palm boiler after the incineration process of oil palm shell and mesocarp fibre. In this study, mortar specimens were prepared to investigate the performance of POC powder. Normal sand with specific gravity 2.60 was utilized as fine aggregate in this study. OPC Type I cement was used as the cementitious material, while POC powder was prepared by grinding POC into a fine powder form. Figure 3 shows a large piece of POC collected from a palm oil mill, and Figure 4 shows the POC fine. Table 2 shows the chemical properties of the materials used in this study. As observed, the silica (SiO_2_) content is on the higher side. Figure 5 shows the particle size distribution curve for the POC powder and cement used in this study. Generally, the POC powder specimens can be considered of similar fineness compared to cement although both have different passing percentages at different size intervals. Table 3 tabulates the particle size distribution of the cement and POC powder. Figure 6 shows the morphology for the POC powder specimens obtained through the scanning electron microscopy (SEM) test. As observed from the figure, the shape of the powder particles is very much irregular and angular. Some of them are cuboidal in shape with sharp edges while others are flaky. There is also a possibility that they will have some impact on the self-compactability properties of the mortar specimens. Figure 7 shows the micrograph of only POC powder with electron dispersive X-ray spectroscopy (EDX) on a smaller magnification. As observed, they are also irregular in shape with notable voids or perforated voids. The EDX results confirm the presence of a high amount of silica (SiO_2_) content within the POC particles, as indicated by the X-ray florescence (XRF) results. Figure 8 shows the POC powder particles at a much smaller magnification with EDX. Despite having irregular and sharp edges, the flat layer surfaces are also evident at the smaller magnification. Figure 9 shows the peaks obtained from analysis of the POC powder through the X-ray diffraction (XRD) test. Aforementioned in Table 2, POC powder is majorly composed of silica (SiO_2_). As observed from the XRD analysis, it is obvious that quartz and cristoballite components, a type of silica compound found to be prominent. Significant sharp peaks and higher peaks were observed at 2θ of 20.83° (quartz), 26.61° (cristoballite), 50.11° (quartz) and 59.93° (quartz). This could affect the blending properties between the cement and POC powder significantly to produce different fresh, hardened and microstructure properties. Figure 10 depicts the quantitative XRD results for POC powder and fly ash. It is worth noting that the amorphous content in these two materials is almost similar.

**Table 2:** Chemical composition of POC powder and cement.

Oxides	POC Powder	Cement
CaO	6.37	64.00
Al_2_O_3_	5.37	5.37
K_2_O	15.10	0.17
MgO	3.13	3.13
SO_3_	2.60	2.61
Na_2_O	0.24	0.24
P_2_O_5_	0.07	0.07
SiO_2_	59.90	20.29
Fe_2_O_3_	6.93	2.94
Mn_2_O_3_	0.12	0.12
TiO_2_	0.12	0.12

**Table 3:** Particle size distribution of cement and POC powder.

Properties	Cement	POC Powder
Average size, D (*v*, 0.5)	27.98 μm	20.97 μm
Passing 10.48 μm (%)	27.58	37.86
Retained 10.48 μm, Passing 48.27 μm (%)	45.80	34.05
Retained 48.27 μm (%)	26.62	28.09

### 2.2. Mix Proportion and Method

A polycarboxylate superplasticizer (SP) with a density of 1.08 g/L was used in this study. In this study, POC powder was replaced with cement between 0% and 50% by binder weight. The mix proportion is shown in Table 4 and it can be seen that the total binder for the replacement mixes are different due to the difference in the specific gravity values for cement and POC powder in order to maintain the same mix volume. The dosage of SP was maintained between 0.5% and 0.8% for this study, while the water binder ratio was fixed at 0.29. The fresh self-compacting mortar properties were evaluated through the slump flow test. Hardened properties studies were carried out to establish the compressive strength, flexural behaviour and water absorption test. In addition, ultrasonic pulse velocity (UPV) test were conducted as part of a non-destructive testing. Besides that, microstructure analysis was also carried out on POC powder and mortar specimens. Mortar cubes 50 mm^3^ in size were prepared for compression testing. The test was carried out according to BS EN 12390-3 [36]. Flexural test was performed using a mortar beam of size 40 mm × 40 mm × 160 mm. The test was performed according to ASTM C348 [37]. Moreover, the water absorption test was carried out on mortar specimens according to the BS 1881-122 [38]. For each value provided in a graph, a minimum of three specimens were tested to ensure consistent and accurate results were obtained.

**Table 4:** Mix proportion for POC powder self-compacting mortar (SCM).

Mix ID	Sand (kg/L)	Cement (kg/L)	POC Powder (kg/L)	W/B	Superplasticizer (SP) Dosage (%)
POC 0	1.14	0.91	0.00	0.29	0.50–0.80
POC 5	1.14	0.86	0.05		
POC 10	1.14	0.81	0.09		
POC 15	1.14	0.76	0.13		
POC 20	1.14	0.71	0.18		
POC 30	1.14	0.62	0.26		
POC 40	1.14	0.52	0.35		
POC 50	1.14	0.43	0.43		

## 3. Results and Discussion

### 3.1. Fresh Properties

**Figure 11:**
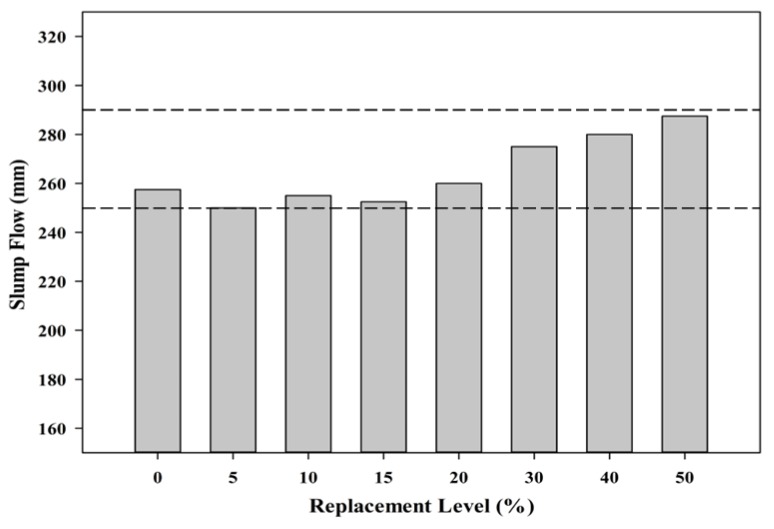
Slump flow results.

The slump flow test provides an insight into the possible stress that exists from the materials that are utilized for mortar production. Self-compacting mortar was specifically chosen to enhance the flow ability of the mixes besides providing good surface finishing. Besides that, it also allows for greater compaction rate which produces dense concrete structure. Figure 11 shows the effect on the slump flow of the substitution with POC powder. The mix proportion was designed to ensure that the fresh properties meets a particular range to ensure compliance with the self-compacting mortar specifications [39]. In this study, the mixes were designed to achieve a slump flow of about 250–290 mm, which is vital to satisfy the self-compactability nature.

### 3.2. Hardened Properties

#### 3.2.1. Compressive Strength

**Figure 12:**
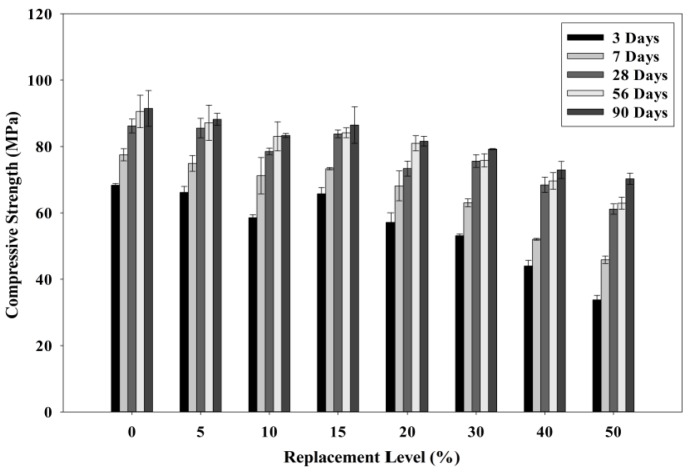
Relationship between POC powder replacement and compressive strength.

**Figure 13:**
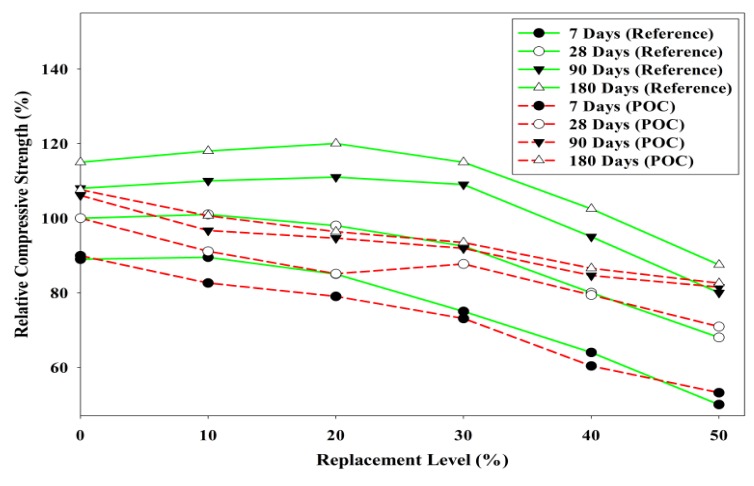
Comparison of relative compressive strength between POC powder and OPC-pozzolan mortar mixes (Adapted from Hewlett [40]).

Figure 12 shows the compressive strength results at different POC powder replacement. Comparing the results up to 90 days, the strength attainment for the POC replacement samples at later stages did not exceed the strength of the control specimens. However, it should be noted that the 50% replacement mix gave a strength value of about 70% of the control mix. Figure 13 depicts the relative compressive strength of POC powder specimens at different replacement levels with a reference study by Hewlett [40] on OPC-pozzolan mortar mixes. It is apparent that the trend of the POC mixes is opposite to the trend compared with the reference mixes. Thus, it supports the dominance of the dilution and filler effects for the POC mixes since there is no significant strength gain as compared to the control mix in this study. In general the optimum cement replacement level with a pozzolanic material is 20%. Thus a comparison with a pozzolanic material such as fly ash at the same replacement level will give some indication of the pozzolanic reactivity of the POC powder. From this study, the late strength gain for 20% POC powder replacement is only about 12% for the period between 28 and 90 days. In comparison, based on literature [41], SCM incorporating 20% fly ash produced approximately 20% strength gain for the same period. Despite having similar amorphous content, the strength pick up for POC powder is considerably lower indicating that it is a weak pozzolan compared to fly ash. If, however, there is significant pozzolanic reaction of the POC powder, a relative strength gain at 28 days and later should be noticeable in the trend giving higher strength values compared to the control. This trend can be observed for other replacement materials which are pozzolanic in nature such as fly ash, rice husk ash and POFA. The early strength achievement for the POC mixes was relatively slow compared to that of the control mix. The dilution effect could be one of the reasons behind the strength loss when POC powder is replaced. For the higher level of POC powder replacement, the availability of excess water from the dilution effect lowers the rate of the hydration process delaying the achievement of strength properties.

**Figure 14:**
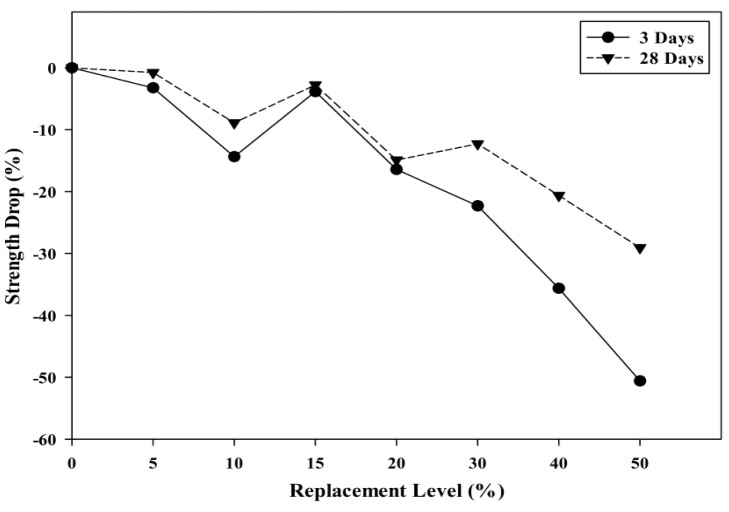
Relationship between POC powder replacement and compressive strength drop.

Figure 14 shows the compressive drop analysis for each POC replacement. For POC 50, at three days of curing, almost 49% strength loss was observed compared to the control mix. The evolution of strength for the POC powder incorporated mixes improved at later ages where at 28 days almost 70% of the strength could be achieved by the POC 50 mix. Researchers have also reported that the availability of a high amount of pores, which results in a state of high permeability could also affect the mortar strength achieved [42]. Again, the poor packing between the cement and the POC powder due to size difference and shape may contribute to the strength loss. In addition, the distribution of POC powder also greatly contributes towards strength loss. According to Mehta [43], cement particles within a range of 10–45 μm delays strength properties while below 10 μm contributes towards early strength. A qualitative assessment of the particle size distribution from Table 3 shows that 37.86% of the POC powder is finer than 10 μm compared to that of cement, which is only 27.58%. At a higher level of replacement, the early strength of the mortar specimens is much lower, which may be due to the highly substituted finer cement particles with inert POC powder. In addition, the strength differences reduce drastically at a later age (28 days), which comes from the lower replacement of cement with POC powder in a range of 10–45 μm. The availability of high cement particles despite the higher replacement rate helps in respect of the late strength achievement.

**Figure 15:**
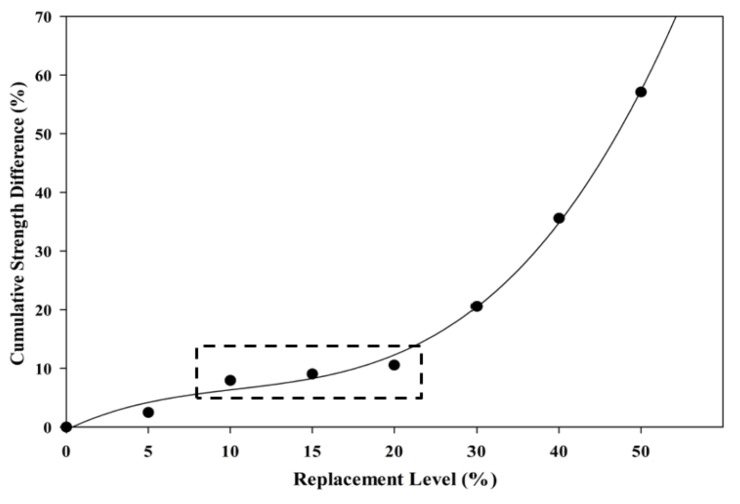
Relationship between cumulative strength difference and POC powder replacement.

Figure 15 shows a plot of the cumulative strength difference between three days and 28 days of hardened specimens against the POC powder replacement level. “Cumulative values of strength” was computed by accumulating the strength differences between three days and 28 days for each replacement level. The cumulative values of the strength difference show the possible replacement level that could provide good strength achievement even though the cement is replaced continuously. As observed, almost identical values in the strength drop between the 10% and 20% replacement level were obtained indicating that the decrease in strength for early age (three days) and later age (28 days) was similar despite increasing the POC powder replacement. It can be deduced that within this replacement level, the combination of cement particle size contributing to the early and later strength is almost identical, thus minimizing the drop in strength. Beyond 20% replacement, the POC powder takes out more of the cement particles of 10 μm and below, and, as a consequence, there is more inert material leading to higher strength loss at an early age.

#### 3.2.2. Ultrasonic Pulse Velocity (UPV)

**Figure 16:**
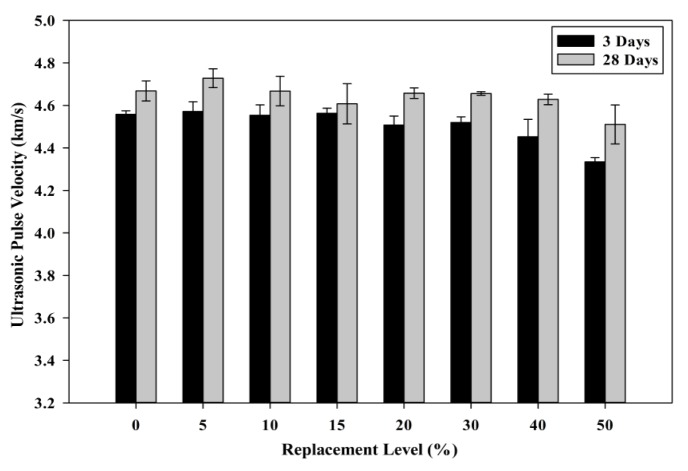
Relationship between ultrasonic pulse velocity (UPV) value and POC powder replacement.

The packing level of the aggregates and paste can be established using the UPV test. Previous studies have mentioned that self-compacting concrete (SCC) usually has a better interfacial transition zone (ITZ) and properly distributed voids within the concrete [44]. Figure 16 shows the relationship between the POC powder replacement level and the UPV values. SCM tends to have a better interface between the aggregate and paste due to its self-compatibility nature whereby they integrate well to form an enhanced structure. The packing level of the aggregate and paste are further enhanced as the amount of voids or empty regions within the mortar specimens are minimized. As observed from the SEM morphology of the POC powder, the irregular shapes of the POC powder may exhibit poor packing capability when replaced with cement particles. The difference in self-compactability properties obtained through POC powder incorporation which was observed through fresh properties may also affect the pulse transfer rate. As aforementioned, the presence of voids due to the POC powder shape may reduce the packing level of the mix to produce lower structural performance. This may be indirectly shown by the slightly lower UPV values for specimens at higher replacement rates. The presence of higher micro voids left by the poor interlocking effect may induce an empty void zone, which may reduce the pulse transfer. In addition, the strength development pattern due to the POC powder can also be observed through the UPV values. Although the reduction in strength for the POC powder samples provides a lower pulse transfer compared to the control specimens, the incorporation of POC powder showed satisfactory results as previous studies reported that UPV values of between 3660 and 4575 m/s can be deemed as “good” quality [45]. As observed from the results obtained, at 28 days all the UPV values was well above 4500 m/s.

#### 3.2.3. Flexural Strength

**Figure 17:**
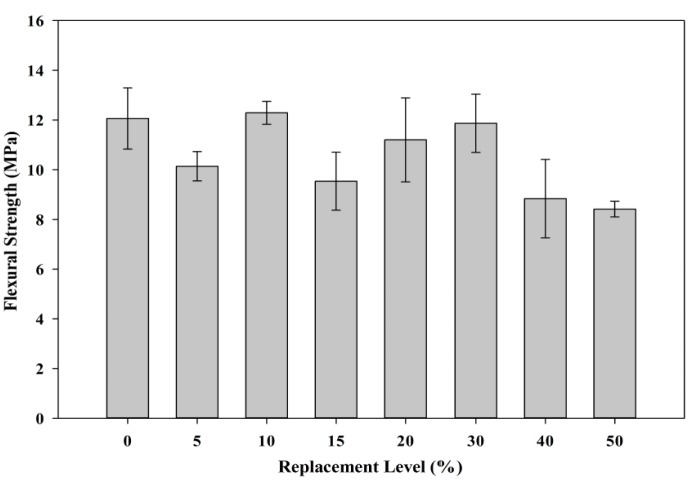
Relationship between POC powder replacement and flexural strength.

Figure 17 shows the relationship between the replacement of POC powder and the flexural strength obtained. The bond between the aggregate with significantly reduced cement content could provide some indication concerning the effectiveness of the powder to act as a binder to enhance aggregate paste interface. This affects the load bearing capacity of the aggregate paste structure. Besides that, the irregular and non-uniform shape of POC powder particles could also provide some effect to the flexural strength achievement. At higher replacement level, the flexural strength is much lower which probably contributed by the poor mortar structure formation due to the presence of POC powder particles. Despite replacing cement with 50% cement, almost 69% of flexural strength can be achieved at maximum replacement (POC 50) relative to control specimens. The SEM test was carried out to investigate the boundary between the aggregate and the binder.

#### 3.2.4. Water Absorption

**Figure 18:**
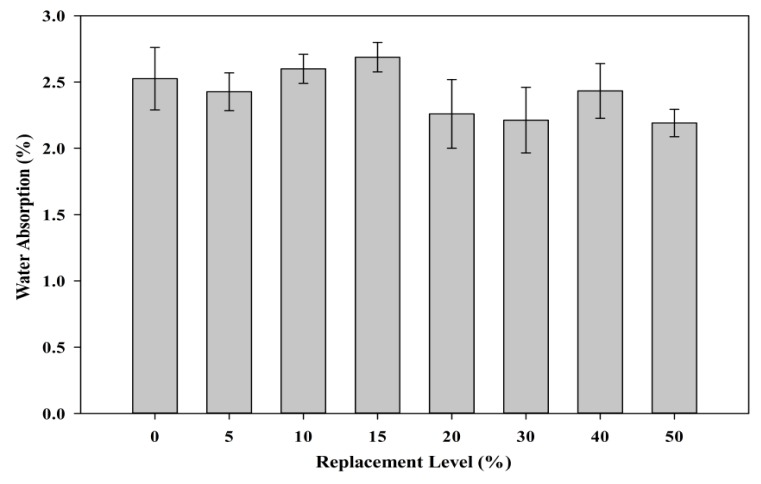
Relationship between POC powder replacement and water absorption.

Figure 18 shows the effect of the POC powder substitution on the water absorption rate of hardened specimens. As observed, different replacement levels produced almost similar water absorption criteria compared to the control specimens. Although the fineness of POC powder and cement slightly varies at different intervals, it probably does not affect the water intake properties. Irregularities in the shape of the POC powder particles could provide a poor interlocking bond to increase the presence of micro voids to give different water absorption characteristics.

#### 3.2.5. Structural Efficiency

**Figure 19:**
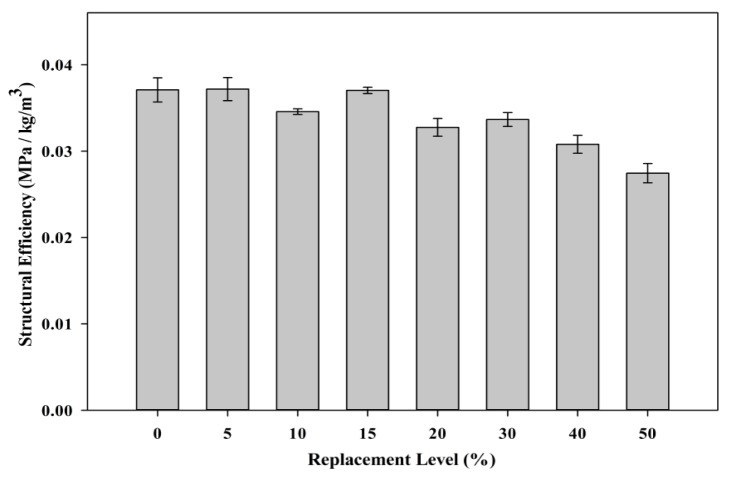
Structural efficiency of mortar specimens.

Figure 19 shows the structural efficiency of the samples incorporating POC powder. The structural efficiency concept was introduced and evaluated to have a similar platform for comparison between POC and non-POC (control) incorporated samples [46]. At a higher replacement level, a reduction in strength was observed as the POC material is acting as a filler material besides having pozzolanicity properties which are rather not significant. The blend of binder between the POC powder and the cement produced a lower strength indicating a poor bond between the cement and the POC powder molecules. In addition, the POC powder acts as an inert material and creates a dilution effect within the hardened mortar structure. Although POC 0 showed significantly better efficiency values, POC powder samples do achieve satisfactory values. At the maximum replacement level, they are able to attain approximately 60% of the structural efficiency compared to the control samples. Integrating both the environmental impact and engineering aspects, the mixes incorporating POC powder definitely provide satisfactory output, which are suitable for application in the construction industry.

#### 3.2.6. Microstructure Analysis

**Figure 20:**
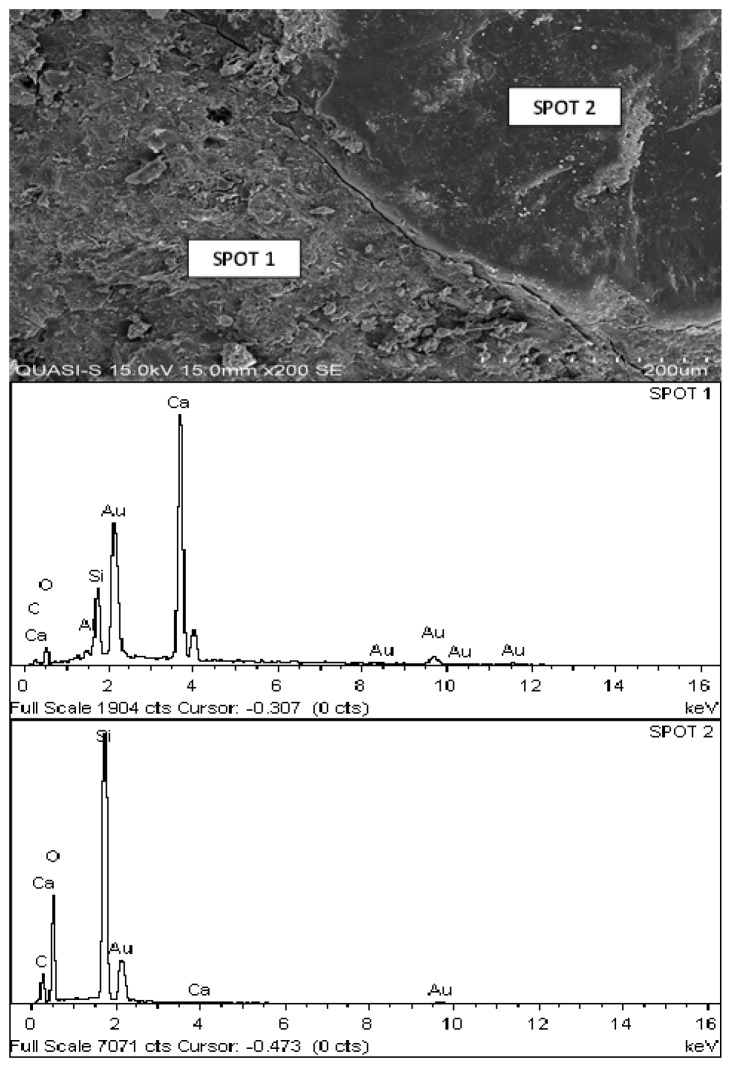
Interface of aggregate and cement paste for POC 0 and POC 40 with EDX.

A SEM study was carried out on each replacement level to investigate the cement paste and aggregate interface. Figure 20 shows the interface between the POC powder and the cement particle confirmed through the EDX results. The self-compactability of the mortar specimens can be observed through the good aggregate paste boundary, which is properly bonded and intact with each other. This is probably due to the high paste volume of SCM. Spot 1 shows the cement paste region while Spot 2 indicates the aggregate as provided by the EDX results. The self-compacting characteristic, which has a high filling ability, can be clearly observed through this figure. The cement paste fills around the aggregate structure to form a strong bond without any detachment. These micrographs are correlated with EDX to investigate the aggregate paste boundary.

### 3.3. Sustainability Performance

#### 3.3.1. Cost Factor

**Figure 21:**
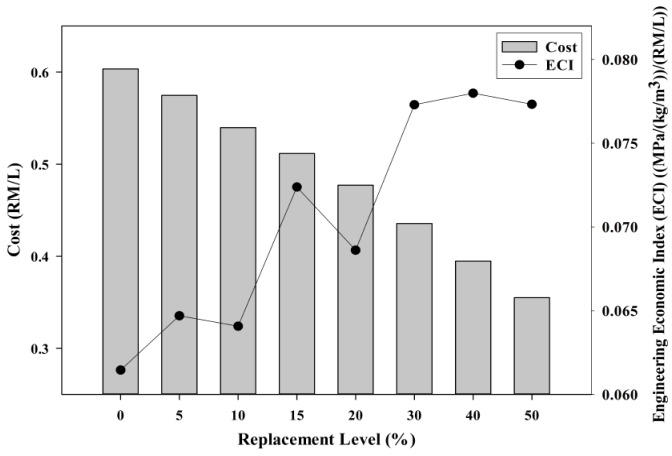
Cost comparison of specimens incorporating POC powder with economic index (ECI).

The cost factor comparison was introduced to evaluate the incorporation of POC powder against cost reduction properties. Figure 21 shows the cost comparison for mortar specimens of different percentages of substitution of POC powder with an engineering economic index (ECI). The cost factors are obtained from previous research conducted by Kanadasan and Abdul Razak [10]. The replacement of POC powder with cement significantly reduces the cost of the concrete whereby almost 41% of the cost can be saved when cement is replaced with 50% POC powder. The cost of POC itself can be considered as “zero” as it is usually disposed of as a waste material without any economic value. The cost for transportation, electricity and manpower are included as part of the POC powder cost assessment study. ECI is a new index developed by integrating the hardened properties of the mortar and the cost factor to provide a quantitative assessment in evaluating the engineering performance with respect to cost factor. ECI is obtained by integrating and computing the ratio between both structural efficiency and cost factor. As discussed earlier in the hardened properties section, the maximum substitution of POC powder also produced satisfactory results to produce medium class strength properties. Hence, when the cost factors are compared with the hardened properties of POC which is an abundantly available waste material and is obtained cheaply, generally it increases the cost efficiency by lowering the overall cost. Intermediate replacement levels produced good engineering to cost comparison values indicating the greater economic potential of POC powder to substitute cement. Certain industries that might need to cut cost could benefit from this type of mortar or concrete specimen without having to compromise on the hardened properties.

#### 3.3.2. Carbon Emissions

**Figure 22:**
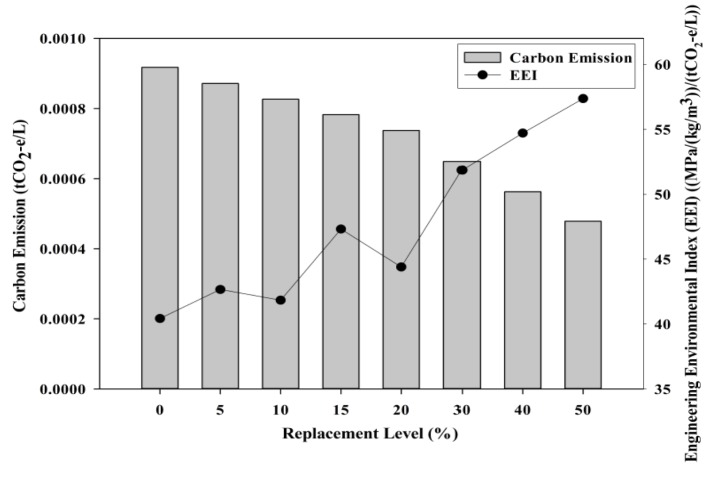
Carbon emissions of specimens incorporating POC powder with engineering environmental index (EEI).

**Figure 23:**
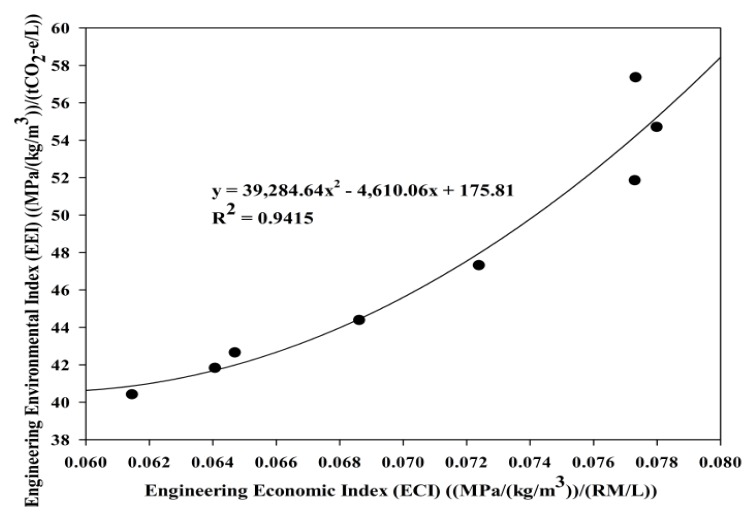
Relationship between ECI and EEI.

The carbon emissions study was incorporated to study the magnitude of the carbon emission reduction when POC powder is utilized as a substitute for cement. Figure 22 shows the effect of POC powder replacement on carbon emissions. The boundary was set to ensure that the transportation of materials is not included within the carbon emission evaluation. POC is completely obtained as a burned final by-product, which, basically, does not involve any further processing stages to transform into some other form. The emission values were obtained through the available research works and database [47,48]. On average, POC samples do produce lower carbon emissions considering the substantially lower emissions for production of POC whereby the CO_2_ footprint for this material is assumed to be zero. As observed, the carbon footprint at the maximum POC powder replacement is lower compared to the control mixes of which a reduction of approximately 52% can be observed when 50% of cement is replaced with POC powder. Considering the structural efficiency, satisfactory hardened properties can be achieved despite the higher substitution level of cement. Besides that, engineering environmental index (EEI) was introduced to assess a new concept that is relating both the engineering and environmental performance of POC powder utilization. It is obtained by computing an analysis on the ratio between structural efficiency and carbon emission values obtained from the test results. POC 50 produced a viable mix, both environmentally and engineering, as it performed better compared to the control specimens in respect to protecting nature from the negative impacts arising from the high utilization of cement. Figure 23 shows the relationship between engineering economic index (ECI) and engineering environmental index (EEI). For high POC powder replacement, environmental performance can be enhanced significantly to produce an output that is environmentally friendly besides cost effective, parallel with the global push towards “green” concrete products. The incorporation of POC powder would not only help to reduce environmental pollution but also improve the waste management system in the palm oil milling industry. The total cement production in Malaysia was 21.46 Mt in 2013 [49]. Based on the biomass production rate reported by Sumathi, Chai and Mohamed [6], replacing cement with POC powder can approximately save 3.3% of cement production annually for every one tonne of CPO produced. This would help to reduce carbon emissions from annual cement production by 3.3%, besides providing an alternative for management of waste generated from palm oil production.

#### 3.3.3. Energy efficiency

Energy savings are becoming vital these days to ensure availability for future use. Consumption can be lowered through the reduction in time and the rate of machine utilization. The energy requirement is significantly lower for the production or preparation of POC powder. POC chunks, which are obtained from palm oil mills, are crushed in the laboratory to produce POC powder. The highly porous nature of POC chunks allows for an easier and faster preparation process. The aggregate crushing value (ACV) gives a good medium to understand qualitatively the possible energy requirement to produce POC powder. As observed from previous study by Kanadasan and Razak [9], the ACV value for gravel is three times higher than for POC aggregate, thereby indicating higher energy consumption. This indirectly indicates that the value of energy consumption can be reduced significantly when POC are processed. The speed of construction can be accelerated concurrently as the time span to prepare the aggregate can be shortened. In addition, the electricity consumption would be much lower, which would promote significant cost savings. This could help to reduce the cost of construction as well as elevate the efficiency of the construction industry.

## 4. Conclusions

To preserve the environment from pollution, the utilization of waste by-products in the construction industry, especially in concrete, would be an ideal choice. From the results of this study, it can be concluded that POC powder is suitable for use as a filler or binder material to replace cement.

(1) It could also reduce the cost of production of medium strength hardened specimens. Despite replacing cement at 50%, POC powder specimens managed to produce almost 70% of the strength when compared to control specimens. Besides that, POC powder samples managed to achieve 60% structural efficiency of that of normal mortar. These satisfactory results indicate suitability of using POC powder for mass concreting works.
(2) Economically, the cost of construction can be reduced without forgoing vital engineering performance. POC powder incorporation managed to reduce the cost of mortar by 41% compared to control specimen. ECI values showed substantial improvement when POC powder is replaced with cement.
(3) The carbon emission was lowered by 52% when POC powder is used. Besides that, EEI values for all replacement levels was higher compared to control specimens indicating the environmental friendliness of the material.
(4) As the world is moving towards the concept of reducing, recycling and reusing (3R), the utilization of POC will not only help to provide an alternative binder material but also help in terms of proper disposal. The exploitation of natural resources can be decreased through the redirection of waste materials in the production stages. This will ensure the availability of natural resources for future generations. The sustainability of the construction industry can be enhanced as there will be an alternative supply of cement instead of using natural resources.
(5) Certain concreting works that require a lower cost but high engineering efficiency may gain advantages. For example, concrete repair and rehabilitation works, which require high flowable mixes and above normal strength, may benefit by utilizing blended cement at a much lower cost. From an environmental perspective, the inclusion of POC powder as a replacement material for cement tends to improve the cost factor, energy usage and ideal emission factor.
